# DYNAPENIA-OBESITY AND PHYSICAL FUNCTION AMONG MEXICAN AMERICAN OLDER ADULTS: NATIVITY DIFFERENCES

**DOI:** 10.1093/geroni/igae098.2605

**Published:** 2024-12-31

**Authors:** Anita Brandt, Soham Al Snih

**Affiliations:** The University of Texas Medical Branch, Galveston, Texas, United States; University of Texas Medical Branch in Galveston, Galveston, Texas, United States

## Abstract

We used data from the Hispanic Established Population for the Epidemiological Study of the Elderly to examine nativity differences in the relationship between dynapenia-obesity and physical function decline over 12-years of follow-up (2004/05-2016) among 767 Mexican Americans aged ≥75 years with a score ≥7 on the Short Physical Performance Battery (SPPB) at baseline. Measures included socio-demographics, medical conditions, body mass index (BMI), depressive symptoms, cognitive function, and disability. Dynapenia was defined as handgrip strength (< 27 Kg in males and < 17 Kg in females). Obesity was defined as BMI ≥30 Kg/m2. Participants were grouped into four groups: No dynapenia – No obesity (n=360), obesity only (n=135), dynapenia only (n=203), and dynapenia-obesity (n=60). Physical function was assessed with the SPPB test. Linear Mixed models were used to estimate the population average change in SPPB as a function of dynapenia-obesity groups by nativity, controlling for all covariates. Participants with dynapenia only experienced a greater (β=-0.75, SE=0.24, p-value=0.0016) decline in physical function compared to those with No dyanpenia – No obesity among US-Born, controlling for all covariates. Participants with dynapenia-obesity group experienced a greater (β=-1.72, SE=0.53, p-value=0.0015) decline in physical function compared to those with No dynapenia – No obesity among foreign-born, controlling for all covariates. In conclusion, the relationship between dynapenia-obesity groups and physical function decline differed by nativity. Dyanpenia-obesity only was associated with decline in physical function among foreign-born. These findings highlight the need to promote healthy weight and improve muscle strength to prevent physical function decline in this population.

